# On the underlying assumptions of threshold Boolean networks as a model for genetic regulatory network behavior

**DOI:** 10.3389/fgene.2013.00263

**Published:** 2013-12-11

**Authors:** Van Tran, Matthew N. McCall, Helene R. McMurray, Anthony Almudevar

**Affiliations:** ^1^Department of Biostatistics and Computational Biology, University of Rochester Medical CenterRochester, NY, USA; ^2^Department of Biomedical Genetics, University of Rochester Medical CenterRochester, NY, USA

**Keywords:** Boolean network, genetic regulatory network, attractor, steady state, state augmentation, asynchronous update, feedback loop, yeast cell-cycle

## Abstract

Boolean networks (BoN) are relatively simple and interpretable models of gene regulatory networks. Specifying these models with fewer parameters while retaining their ability to describe complex regulatory relationships is an ongoing methodological challenge. Additionally, extending these models to incorporate variable gene decay rates, asynchronous gene response, and synergistic regulation while maintaining their Markovian nature increases the applicability of these models to genetic regulatory networks (GRN). We explore a previously-proposed class of BoNs characterized by linear threshold functions, which we refer to as *threshold Boolean networks* (TBN). Compared to traditional BoNs with unconstrained transition functions, these models require far fewer parameters and offer a more direct interpretation. However, the functional form of a TBN does result in a reduction in the regulatory relationships which can be modeled. We show that TBNs can be readily extended to permit self-degradation, with explicitly modeled degradation rates. We note that the introduction of variable degradation compromises the Markovian property fundamental to BoN models but show that a simple state augmentation procedure restores their Markovian nature. Next, we study the effect of assumptions regarding self-degradation on the set of possible steady states. Our findings are captured in two theorems relating self-degradation and regulatory feedback to the steady state behavior of a TBN. Finally, we explore assumptions of synchronous gene response and asynergistic regulation and show that TBNs can be easily extended to relax these assumptions. Applying our methods to the budding yeast cell-cycle network revealed that although the network is complex, its steady state is simplified by the presence of self-degradation and lack of purely positive regulatory cycles.

## 1. Introduction

Dynamic models are used frequently to study the evolution of a genetic regulatory network (GRN) over time [see De Jong (2002) for a review]. Often accompanying these models is a graph representing the relationships among the genetic components (e.g., proteins, DNA, RNA). The components are represented by nodes and the regulatory relationships by edges. The dynamic models range from highly quantitative frameworks such as systems of differential equations [see Heinrich and Schuster (1996) for an introduction] to more qualitative models such as Boolean networks (BoN) (Kauffman, 1969). Although systems of differential equations are explicit and detailed in their description of network trajectories, they require specialized knowledge of kinetic parameters, time constants, and the mechanism underlying the process. In comparison, BoN are easier to construct and interpret. In a BoN, gene expression is discretized into one of two states, e.g., on/off, up/down, or active/inactive. Regulation is modeled by logic functions (e.g., AND, OR, NOT) that code the influence of the effector genes. Genetic regulation is either positive, resulting in increased gene expression, or negative, resulting in decreased gene expression. While discretizing gene expression is certainly a simplification, similar approaches have resulted in increased reproducibility and robustness when estimating both absolute and differential gene expression (Parmigiani et al., 2002; Scharpf et al., 2003; Zilliox and Irizarry, 2007; McCall et al., 2011), and Boolean network models have been used to successfully model gene regulatory networks (Albert and Othmer, 2003; Espinosa-Soto et al., 2004; Li et al., 2004; Davidich and Bornholdt, 2008). For certain small networks, systems of differential equations and BoN are qualitatively similar in their state transitions and long term behavior (Glass and Kauffman, 1972, 1973). These two types of models can differ in their results when applied to networks with many nodes and complex gene interactions.

Ultimately a desirable model is one that retains the relative ease of modeling and interpretation of a BoN and the quantitative precision of differential equations. A model that possesses these qualities is the BoN proposed by Li et al. (2004) to study the budding yeast cell-cycle. Cited by more than 600 articles, their BoN employs a simple, elegant linear function with a threshold that utilizes far fewer parameters than a BoN specified by truth tables. Because of the influential results of Li et al.'s threshold Boolean network (TBN) model, a thorough analysis of the model's mathematical properties and fidelity to true network behavior are important. A key aspect of their model is the treatment of genetic degradation. Degradation primarily occurs in three ways: (a) negative regulation by other genes in the network, (b) negative regulation by other (unmeasured) genes not in the network, and (c) intrinsic protein degradation. The latter two are indistinguishable in a GRN and are commonly referred to as *self-degradation*.

**Network Inference**

- Q: Which kinds of biological networks have been inferred in the paper?
- A: We studied genetic regulatory networks (GRN), specifically the budding yeast cell-cycle network, using a threshold Boolean network (TBN) model specified by linear functions and a threshold.
- Q: How was the quality/utility of the inferred networks assessed? How were these networks validated?
- A: We studied how the TBN model behaves under different assumptions of gene self-degradation and different parameter specifications. We Markovianized self-degradation and showed that the resulting model is more tractable. We proposed and proved two theorems relating gene self-degradation to a TBN's attractor set and used these results to assess the behavior of the budding yeast cell cycle. Our results were then compared to those of a widely cited GRN model.
- Q: A few sentences explaining the main positive/negative results described in the paper.
- A: We showed how the TBN model accommodates aspects of GRNs such as variable Markovian self-degradation, asynchronous gene update, and synergistic relationships, making the model more representative of real biological networks. Additionally, we found that the complexity of a GRN can be summarized by the presence of self-degradation and cycles comprised of only positive regulations. The primary limitation of TBNs is that they cannot easily model all possible regulatory relationships. Nevertheless, the mathematical tractability and qualitative characteristics of a TBN make it a desirable model for understanding GRNs.

Our evaluation of the TBN consists of: (1) characterizing the regulatory relationships that the TBN can and cannot express, (2) showing how self-degradation has a substantial impact on a GRN's steady state behavior, (3) Markovianizing self-degradation, (4) proving that steady states of a GRN are sensitive to gene interaction strengths, (5) commenting on the role of self-degradation and interaction strength in asynchronous gene update, and (6) augmenting the TBN to allow for synergistic and antagonistic relationships. The extensions improve a TBN's representation of a GRN and the theoretical results break down its complexity. In Section 2, we formally introduce BoN, their dynamic properties and Li et al.'s cell-cycle TBN. In Section 3, we evaluate the TBN and present our theorems relating self-degradation to steady state behavior. A summary and discussion of our findings follows in Section 4.

## 2. Materials and methods

### 2.1. A review of boolean networks and dynamic properties

A Boolean Network (BoN) is defined as a directed graph 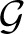
(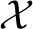, 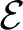) with Boolean transition functions. The graph 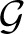 is composed of a set of nodes 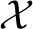 = {1, …, *N*} and a set of edges 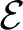, in which a directed edge represents a causal relationship between two nodes. Each node *i* can have either state *x*_*i*_ = 0 or *x*_*i*_ = 1. Whenever there is an edge *i* → *j* ∈ 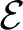, *j* is called the *child* of *i* and *i* is called the *parent* of *j* in 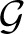. Associated with each node is a Boolean function *f*_*i*_: 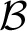
^*N*^ ↦ 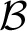 where 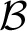 = {0, 1}. This function specifies how the state of node *i* changes over time. Denote the state of node *i* at time *t* as *x*_*i*_(*t*). Node *i* updates its state by the Markovian process, *x*_*i*_(*t* + 1) = *f*_*i*_(*x*_1_(*t*), …, *x*_*k*_(*t*)) where 1, …, *k* are its parents. In other words, the current state of a node is determined by a function of its parents' previous states. Although *f*_*i*_ is defined to take *N* inputs, the relevant arguments are the parents' states since all other nodes do not directly affect *i*. In GRNs, an *f*_*i*_ specifies the regulatory relationship between gene *i* and the rest of the network. The entire network updates synchronously by the process, **x**(*t* + 1) = *A*(**x**(*t*)), where **x** = (*x*_1_, …, *x*_*N*_) is a state vector and *A*: 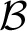
^*N*^ ↦ 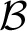
^*N*^ is the model's operator. To be exact, *A* is a vector whose components are the functions, *f*_*i*_. A network path is a sequence,

x(0)→x(1)→x(2)→…

The long term behavior or steady state of a BoN can be characterized by its attractors. An *attractor* is a set of network states that occur infinitely often in the sequence *A*^*t*^(**x**(0)) with t ≥ 1. If the set contains only one element, then the attractor is referred to as a fixed point, otherwise the attractor is periodic. Formally, a *fixed point* is defined as **x** = *A*(**x**). An important feature of an attractor is its *basin of attraction*, which is the set of state vectors from which the network reaches the attractor. The size of the basin of attraction represents the attractor's pull on the network states. Growing evidence suggests that an attractor represents a particular cell fate (Kauffman, 1969; Huang et al., 2005).

### 2.2. The cell-cycle threshold boolean network

The cell-cycle of the budding yeast *Saccharomyces cerevisiae* is a phenomenon that continues to fascinate and generate knowledge even after years of research. Li et al. (2004) developed a dynamic BoN to model the cycle and “demonstrated that the cell-cycle network is extremely stable and robust for its function” (p.4781). Their BoN uses a linear transition function with a threshold, henceforth referred to as a TBN, in the following manner:

(1) $$x_{i}(t+1)=\{\begin{array}{ll} \;1, & \sum_{j}a_{ij}x_{j}(t)>0\\ \;0, & \sum_{j}a_{ij}x_{j}(t)<0\\ x_{i}(t), & \sum_{j}a_{ij}x_{j}(t)=0 \end{array}$$

where *x*_*j*_(*t*) is the expression of the regulator protein *j* at the current time *t*, *x*_*i*_(*t* + 1) is the expression of the regulated protein *i* at the next time *t* + 1, and interaction coefficient *a*_*ij*_ codes the strength and type of regulation that protein *j* exerts on protein *i*. Positive regulation is specified by positive values of *a*_*ij*_ and negative regulation by negative values of *a*_*ij*_. Any regulation is a product of the parent's state *x*_*j*_(*t*) and the type and strength of the regulation *a*_*ij*_. The next state of a protein depends only on its parents' current states. Specifically, the next state *x*_*i*_(*t* + 1) of protein *i* is ‘on’ if the sum of its parents' regulatory effects surpasses 0, “off” if the sum is below 0, and when the sum is 0, the state remains the same. *Self-degradation* is a process not incorporated in Equation (1), but defined separately as: if ∑_*j*_ *a*_*ij*_*x*_*j*_(*t*) = 0 from *t* = *t*_*s*_ to *t* = *t*_*s*_ + *t*_*d*_ − 1 then *x*_*i*_(*t*_*s*_ + *t*_*d*_) = 0, where *t*_*d*_ is referred to as the protein's *lifetime*. A higher value of *t*_*d*_ translates to a slower rate of decay. In the cell cycle TBN constructed in Li et al. (2004), only proteins not negatively regulated by others possess the self-degradation property (we note, however, that Swi5 appears to be an exception, as indicated in Figure 1 of Li et al. (2004)). Proteins that do not self-degrade maintain their current state according to line 3 of Equation (1). For ease of reference, we refer to these proteins as having the *persistence* property.

Proteins in the cell-cycle network belong to one of four classes: (a) cyclins (Cln1,-2,-3, Clb1,-2,-5,-6), (b) inhibitors/competitors of cyclins (Sic1, Cdh1, Cdc20, Cdc14), (c) transcription factors (SBF, MBF, Mcm1/SFF, Swi5), and (d) checkpoints. We focus on a simplified network having only the cell size checkpoint. The cell-cycle starts at phase G1 where the cell size becomes large enough and Cln3 reaches a high enough concentration, i.e., its Boolean state is equal to 1. When these two conditions are met, the cell commits to division. Next, the cell moves into S phase in which DNA is synthesized. After S phase is the gap phase G2, and in the final phase M, chromosomes separate and the yeast cell divides into two cells. This phenomenon repeats when the right conditions encourage cell growth and division.

**Figure 1:**
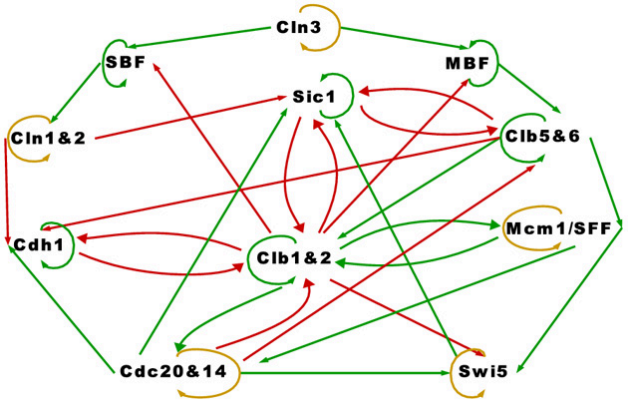
**The simplified yeast cell-cycle network**.

Accompanying the TBN model in Equation (1) is a graph depicting the relationships among the proteins in the cell-cycle network. We reproduced the cell-cycle network in Figure 1. The graph is identical to Li et al.'s except for green self loops that we added to proteins that are assumed to persist. Functionally, Figure 1 is equivalent to theirs. An edge between two nodes represent one of four regulatory relationships, negative regulation, positive regulation, self-degradation and persistence. These relationships are represented with a *red edge*, *green edge*, *yellow loop*, and *green self loop* respectively (note that all genes possess either a green self loop or a yellow loop). Li et al. assigned all positive regulations (green edges) the same interaction coefficient *a*_*ij*_ = *a*_*g*_, and all negative regulations (red edges) *a*_*ij*_ = *a*_*r*_. Although *a*_*ij*_ is allowed to take on any real value, Li et al.'s main results are based on *a*_*g*_ = −*a*_*r*_ = 1. They claimed that “the results are insensitive to the values of the weights *a*_*g*_ and *a*_*r*_ … and to the protein lifetime *t*_*d*_, as long as −*a*_*r*_ ≥ *a*_*g*_ and *t*_*d*_ > 0” (p. 4785).

The cell-cycle network in Figure 1 appears to be very complex. The network contains 11 proteins, some proteins have as many as five regulators, and there are many feedback loops. With the exception of Swi5, a protein that is not negatively regulated by others in the network self-degrades (yellow loop), otherwise it persists (green self loop). We will show how the attractor set changes when Swi5 is set to persist instead of degrade, which illustrates the network's sensitivity to the assumptions of self-degradation. An important feature of this network is that the positive regulations (green edges) are almost acyclic except for the cycle between Clb1&2 and Mcm1/SFF, key players in the M phase or mitosis. We will discuss in more detail how this cycle plays a crucial role in the simplicity of the network's long term behavior.

Compared to a BoN specified by truth tables, the TBN in Equation (1) captures genetic relationships with far fewer parameters, which is especially convenient when the model space is relatively large. As an illustration, suppose a network has *N* nodes and each node *i* has *k*_*i*_ parents. Defining a BoN with truth tables requires $\sum_{i}^{N}2^{k_{i}}$ parameters, 2^*k*_*i*_^ parameters per node, while specifying the TBN in Equation (1) requires only $\sum_{i}^{N}k_{i}$ parameters, *k*_*i*_ of *a*_*ij*_ per node. The TBN is a hybrid between a BoN and a system of differential equations that retains the interpretability of the former and the mathematical tractability of the latter.

In the next section, we analyze the TBN model and propose extensions related to self-degradation, asynchronous gene update and synergistic relationships. We also state theoretical results that translate self-degradation and network cycles to network steady state behavior.

## 3. Results

### 3.1. Threshold boolean network model

The primary limitation of the model described by Equation (1) is that only the regulatory relationship OR can be expressed. For example, given proteins, *i*, *j*, and *k*, expressing *i* if *j* ∪ *k* can be achieved by setting *a*_*ij*_ = *a*_*ik*_ = 1. However, expressing *i* if *j* ∩ *k* is impossible with any combinations of *a*_*ij*_ and *a*_*ik*_. To encode an AND relationship and other types of regulations, the threshold needs to be greater than zero. An example of a TBN with a non-zero threshold was implemented by Davidich and Bornholdt (2008) to model the fission yeast cell-cycle. We present a more general form of the model in Equation (1) by including a threshold parameter α_*i*_ ≥ 0:

(2) $$x_{i}(t+1)=\{\begin{array}{ll} \;1, & \sum_{j}a_{ij}x_{j}(t)>\alpha_{i}\\ \;0, & \sum_{j}a_{ij}x_{j}(t)<\alpha_{i}\\ x_{i}(t), & \sum_{j}a_{ij}x_{j}(t)=\alpha_{i}. \end{array}$$

Clearly, Equation 1 is a special case of Equation 2 in which α_*i*_ = 0 ∀ *i*. By varying thresholds and interaction coefficients, it is possible to encode many regulatory relationships. Given proteins, *i*, *j*, and *k*, encoding the relationship *i* if *j* ∩ *k* would simply require setting *a*_*ij*_ = *a*_*ik*_ = 0.5 and α_*i*_ = 0.99. Even more complicated relationships can be expressed using the TBN model. For example, *i* if (*j* ∪ *k*) ∩ *l* could be achieved by setting *a*_*ij*_ = *a*_*ik*_ = 0.1, *a*_*il*_ = 0.95, and α_*i*_ = 1.

However, not all relationships can be expressed. One such relationship is *i* if (*j* ∩ *k*) ∪ (*l* ∩ *m*). The following example illustrates this issue:

***Example.*** In order to encode the relationship *i* if (*j* ∩ *k*) ∪ (*l* ∩ *m*), the coefficients *a*_*ij*_, *a*_*ik*_, *a*_*il*_, *a*_*im*_ and the threshold α_*i*_ would have to satisfy the following inequalities:

$$\begin{array}{l} a_{ij}+a_{ik}>\alpha_{i}\\ a_{il}+a_{im}>\alpha_{i}\\ a_{ij}+a_{il}\leq \alpha_{i}\\ a_{ij}+a_{im}\leq \alpha_{i}\\ a_{ik}+a_{il}\leq \alpha_{i}\\ a_{ik}+a_{im}\leq \alpha_{i}. \end{array}$$

Summing the first 2 inequalities produces *a*_*ij*_ + *a*_*ik*_ + *a*_*il*_ + *a*_*im*_ > 2α_*i*_. Summing the last four inequalities produces 2*a*_*ij*_ + 2*a*_*ik*_ + 2*a*_*il*_ + 2*a*_*im*_ ≤ 4α_*i*_. The contradiction shows that it is not possible to encode the above relationship using any TBN of the form in Equation (2). Although inclusion of the threshold parameter α_*i*_ permits a far wider range of regulatory relationships, some limitations remain.

### 3.2. Self-degradation

#### 3.2.1. Steady state characteristics

Setting negative regulations (red edges) at the same rate *a*_*ij*_ = *a*_*r*_ = −1, positive regulations (green edges) at the same rate *a*_*ij*_ = *a*_*g*_ = 1 and protein lifetime *t*_*d*_ = 1, the main result of the cell-cycle TBN, reported in Li et al. (2004), is the set of attractors in Table 1A. The largest basin of attraction shown is 1764. Of 2^11^ = 2048 possible network states, 1764 states flow toward the fixed point (0, 0, 0, 0, 0, 0, 1, 0, 0, 1, 0), in which inhibitor proteins Cdh1 and Sic1 stay active indefinitely even when the rest of the network is off. Although the cell-cycle network is very complex, the attractor set has only seven attractors, which are all fixed points.

**Table 1:** **The attractor set for the cell-cycle threshold Boolean network under different interaction coefficients**.

**Basin size**	**Cln3**	**MBF**	**Clb5&6**	**Mcm1/SFF**	**Swi5**	**Cdc20&14**	**Cdh1**	**Cln1&2**	**SBF**	**Sic1**	**Clb1&2**
**(A) *a*_*g*_ = 1**											
1764	0	0	0	0	0	0	1	0	0	1	0
151	0	0	0	0	0	0	0	1	1	0	0
109	0	1	0	0	0	0	1	0	0	1	0
9	0	0	0	0	0	0	0	0	0	1	0
7	0	1	0	0	0	0	0	0	0	1	0
7	0	0	0	0	0	0	0	0	0	0	0
1	0	0	0	0	0	0	1	0	0	0	0
**(B) *a*_*g*_ = 2**											
1978	0	0	0	0	0	0	1	0	0	1	0
57	0	0	0	0	0	0	0	1	1	0	0
7	0	0	0	0	0	0	0	0	0	1	0
5	0	0	0	0	0	0	0	0	0	0	0
1	0	0	0	0	0	0	1	0	0	0	0
**(C) *a*_*g*_ = 3**											
1936	0	0	0	1	1	1	1	0	0	1	1
59	0	0	0	0	0	0	1	0	0	1	0
40	0	0	0	0	0	0	0	1	1	0	0
7	0	0	0	0	0	0	0	0	0	1	0
5	0	0	0	0	0	0	0	0	0	0	0
1	0	0	0	0	0	0	1	0	0	0	0

Protein lifetime is set at t_d_ = 1. All negative regulations are assigned a common coefficient a_ij_ = a_r_ = −1. All positive regulations are assigned a_ij_ = a_g_. (A) Shows the attractor set associated with a_g_ = 1. (B) Shows the attractor set associated with a_g_ = 2. (C) Shows the attractor set associated with a_g_ = 3. For each panel, the rows are the attractors, which are all fixed points, and columns 2 through 12 indicate whether a protein is on (1) or off (0) in the attractor. Column 1 lists the basin size of each attractor.

Thomas (1981) explored the effects of different *regulatory circuits* or feedback loops on the composition of the attractor set. Regulatory circuits are classified as positive or negative depending on whether the number of negative regulations (red edges) in the circuit is odd or even. Thomas proposed that positive circuits are necessary to generate multiple attractors and negative circuits are necessary to generate fixed points and periodic attractors. These ideas were later formalized in theorems by Remy et al. (2008); Richard (2010), and various conditions for a unique fixed point attractor set have been developed by Robert (1980); Shih and Dong (2005); Richard (2013). The theorems and results in this manuscript build upon these works by examining the effect of self-degradation and regulatory circuits on a network's long term behavior.

**Theorem 1**. *Let* 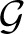 = (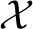, 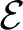) *be a TBN of the form in Equation* (2) *with N nodes*, 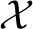 = {1, …, *N*} *and edges* 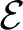. *Suppose each threshold parameter satisfies α_i_ ≥ 0 for each i. If every node has a self-degradation loop and network cycles must have at least 1 negative regulation (red edge), then the network's attractor is a unique fixed point, the null state*.

The proof requires the following definition. Let 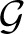 = (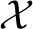, 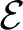) be a graph. An ordering of nodes 1, …, *N* is a *topological ordering* relative to 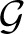 if, whenever we have *i* → *j* ∈ 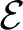, then *i* < *j*. A parent node has a lower order than a child node. Most importantly, a graph is directed acyclic or *DAG* if and only if it has a topological ordering.

***Proof.*** Denote the set of nodes having either an incoming or outgoing positive regulation (green edge) as 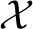
_*n*_ = {1, …, *n*} ⊂ 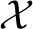. Given that cycles with all positive regulation (green edges) do not exist, choose a topological ordering (with respect to green edges only) for 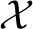
_*n*_, say 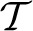, and add directed null edges, which have no real regulatory effect, to all pairs of nodes in 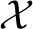
_*n*_ not having an edge such that 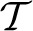 is not violated. Then 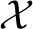
_*n*_ has the unique topological ordering 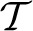 = 1, …, *n*. The expression of a node in 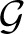 = (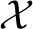, 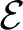) at time *t* is a function of nodes with smaller topological order and other nodes in 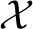 at the previous time *t* − 1, i.e.,

$$\begin{array}{l} x_{i}(t)=f_{i}(\{x_{1}(t-1),\ldots ,x_{i\;-\;1}(t-1)\},\{x_{i}(t-1),\ldots ,\\ \;x_{n}(t-1),\ldots ,x_{N}(t-1)\}) \end{array}$$

where *f*_*i*_ is the transition function for node *i* of the form in Equation (2) in which the parameter *a*_*ij*_ can take any magnitude so long as positive regulation is defined by a positive sign and negative regulation by a negative sign.

The proof proceeds from the observation that, under the stated hypothesis, if for *t*_*d*_ consecutive time points all nodes with topological ordering smaller than *i* have value 0, at the time point *t* immediately following we must also have *x*_*i*_(*t*) = 0.

By mathematical induction, we will show that (*x*_1_(*k*), …, *x*_*n*_(*k*)) = (0, …, 0) for some time *k* and remains at $\vec{0}$ after time *k*. At some time *t* < *k*,

$$\begin{array}{l} x_{1}(t)=f_{1}(\{\emptyset \},\{x_{1}(t-1),\ldots ,x_{N}(t-1)\})\\ \;=0, \end{array}$$

and remains at 0 indefinitely through negative regulation or self-degradation. At some *t*′ > *t*,

$$\begin{array}{l} x_{2}(t^{\prime })=f_{2}^{\left(t^{\prime }-t\right)}(\{x_{1}(t)\},\{x_{2}(t),\ldots ,x_{n}(t),\ldots ,x_{N}(t)\})\\ \;=f_{2}^{\left(t^{\prime }-t\right)}(\{0\},\{x_{2}(t),\ldots ,x_{n}(t),\ldots ,x_{N}(t)\})\\ \;=0 \end{array}$$

where the composite function *f*^(*t*′ − *t*)^_2_ is the (*t*′ − *t*)th iteration of the transition function *f*_2_, and (*t*′ − *t*) ≤ *t*_*d*_, for any *t*_*d*_. Node 2 remains at 0 indefinitely through negative regulation or self-degradation. Assume that for some *l* nodes, all with order less than *n*, satisfies at time *t*″ > *t*′,

$$x_{1}(t^{\prime \prime })=\ldots =x_{l}(t^{\prime \prime })=0,$$

and remains at 0 indefinitely through negative regulation or self-degradation.

Then at time *k* > *t*″,

$$\begin{array}{l} x_{n}(k)=f_{n}^{\left(k-t^{\prime \prime }\right)}(\{x_{1}(t^{\prime \prime }),\ldots ,x_{n-1}(t^{\prime \prime })\},\{x_{n}(t^{\prime \prime }),\ldots ,x_{N}(t^{\prime \prime })\})\\ \;=f_{n}^{\left(k-t^{\prime \prime }\right)}(\{0,\ldots ,0\},\{x_{n}(t^{\prime \prime }),\ldots ,x_{N}(t^{\prime \prime })\})\\ \;=0. \end{array}$$

where *f*^(*k*−*t*″)^_*n*_ is the (*k* − *t*″)th iteration of the transition function *f*_*n*_, and (*k* − *t*″) ≤ *t*_*d*_, for any *t*_*d*_. Node *n* remains at 0 indefinitely. For all nodes not in 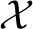
_*n*_, they remain at state 0 through negative regulation or self-degradation. Therefore, (*x*_*i*_(*k*), …, *x*_*N*_(*k*)) = $\vec{0}$ and remains a fixed point after time *k*.     □

**Figure 2:**
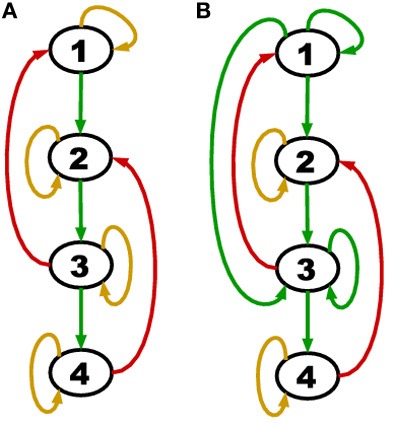
**(A)** A network with all genes self degrading (yellow loop on each node) and acyclic positive regulations (green edges). **(B)** A network with persistence (green self loop) in addition to self-degradation and acyclic positive regulations.

In short, the proof shows that when upstream positive regulations are shut down by self-degradation, the network turns off in a cascading fashion due to the topological order and self-degradation. The theorem applies to an entire class of networks whose member graphs may have any number of genes, any number of cycles with at least one negative regulation (red edge), differing interaction coefficients *a*_*ij*_ and differing protein lifetimes *t*_*d*_. The theorem is invariant to *a*_*ij*_ and *t*_*d*_ because these parameters only work to speed up or slow down the rate at which the network reaches the null attractor. An example of a network belonging to this class is displayed in Figure 2A.

Consider a more general network class that is still acyclic in the positive regulations (green edges) but has the additional feature of persistence (green self loops). An example of such a network is shown in Figure 2B.

We noted above that the degradation model defined here implies an assignment to each gene of either a yellow loop or a green self loop. Theorem 1 concerns the special case in which all genes are assigned yellow loops. A green self loop is formally a cycle (which does not contain a red edge), and so the hypothesis of Theorem 1 does not hold if any persistent nodes are present.

However, suppose we are given a TBN which does satisfy the hypothesis of Theorem 1, but we then alter the model by designating a set of nodes as persistent, otherwise leaving the model unchanged. We wish to determine how this affects the complexity of the resulting attractor structure. It must have some effect. To take a trivial case, suppose we have *n* unconnected persistent nodes. Each may be analyzed as an independent TBN, each of which can sustain a fixed point of value 0 or 1. The total number of unique fixed points for the entire network is therefore 2^*n*^. Of course, the complexity of the attractor structure in this case is due entirely to the lack of any exogenous degradation pathways, and not to any connectivity structure of the network (which does not exist in our example).

We next show that this type of reasoning can be extended to TBNs which have the type of acyclicity defined by Theorem 1, but which also have persistent nodes. It is possible to describe mathematically weaker properties of acyclicity within cyclic networks in a way which bounds the complexity of attractor structure. For example, Skodawessely and Klemm (2011) found the maximum number of fixed points in such a network to be 2^|*V*|^ where *V* ⊆ *N* is a set of nodes whose removal leaves the network acyclic.

Here, we extend our notion of acyclicity in the following way. We say *j* is an *ancestor* of *i* if there is a directed path from *j* to *i*. Define the two sets of nodes:

(3) $$\begin{array}{l} S_{G}=\{\text{ all persistent nodes }\}\\ S_{A}=\{\text{ all nonpersistent nodes not possessing}\\ \text{ a persistent node as an ancestor}\}. \end{array}$$

**Theorem 2**. *Suppose we are given a TBN in which the subnetwork defined by the nodes *S*_*A*_ of* (3) *satisfies the hypothesis of Theorem 1, or for which *S*_*A*_* = ∅.

*Next, define the following sequence of subsets of nodes:*

$$\begin{array}{l} E_{1}=S_{G}\cup S_{A},\\ E_{j}=\{all\;nodes\;not\;in\;\cup_{i<j}E_{i}\;with\;all\;parents\;in\cup_{i<j}E_{i}\},j>1, \end{array}$$

*and suppose for some J all nodes are included in ∪_i≤J_E_i_. Then any two fixed points with identical values for the persistent nodes must be equal, and therefore the maximum number of fixed points is 2^g^, where g is the number of persistent nodes*.

*Proof.* Suppose we are given any fixed point. The nodes in *S*_*A*_ (if any) form a TBN satisfying the hypothesis of Theorem 1, so any fixed point must be 0 on these nodes. This implies that the fixed point values of the nodes in *E*_2_ are determined entirely by those of *S*_*G*_. The argument may be repeated for *E*_3_, *E*_4_, …, until the fixed point values of all nodes are determined.     □

Theorem 2 complements the result of Skodawessely and Klemm (2011). The conclusion implies a similar upper bound of 2^*g*^ for the number of distinct fixed points, where *g* is the number of persistent nodes. However, while the class of BoNs considered by Theorem 2 is more restricted, removal of the persistent nodes does not necessarily leave the network acyclic, so that the result of Skodawessely and Klemm (2011) does not imply Theorem 2.

The hypothesis of Theorem 2 is satisfied by both TBNs of Figure 2. In particular, for **(B)** we have *S*_*G*_ = {1, 3}, *S*_*A*_ = ∅, *E*_2_ = {4}, *E*_3_ = {2}. However, if a negative regulation from node 2 to node 4 was added, the hypothesis would no longer hold (we would have *E*_*j*_ = ∅ for all *j* ≥ 2) and a counter-example could be constructed.

Next, consider, the cell-cycle network of Figure 1. This TBN satisfies the hypothesis of Theorem 2 by setting

$$\begin{array}{l} S_{G}=\{MBF,Clb5\text{\&}6,Cdh1,SBF,Sic1,Clb1\text{\&}2\}\\ S_{A}=\{Cln3\}\\ E_{2}=\{Mcm1/SFF,Cln1\text{\&}2\}\\ E_{3}=\{Cdc20\text{\&}14\}\\ E_{4}=\{Swi5\}. \end{array}$$

It is interesting to note that the hypothesis of Theorem 2 is satisfied despite the existence of a cycle of green edges between Mcm1/SFF and Clb1&2 (due the the fact that one of these nodes is persistent).

We can see from the application of Theorem 2 to the cell-cycle network that the relationship between the attractor structure and the configuration of persistent nodes is similar to the previous example of the completely unconnected TBN, in the sense that all fixed points are fully determined by their values on the persistent nodes, so that the complexity of the attractor structure must be understood to be driven by a selective lack of exogenous degradation pathways.

#### 3.2.2. Self-degradation assumptions

The assignment of self-degradation (yellow loops) to certain proteins in a network is not a trivial task and cannot be completed *ad-hoc* because self-degradation influences the network's long term behavior. The simplicity of the attractor set associated with the cell-cycle network in Table 1A is attributable to the presence of self-degradation and a lack of active network cycles composed entirely of positive regulations (green edges). We exemplify this claim with protein Swi5, the transcription factor for inhibitor protein Sic1. According to Li et al.'s rule of assigning self degradation only to proteins without negative regulators (incoming red edges), Swi5 should not self-degrade since it has the inhibitor Clb1&2. However, their representation of the network allowed Swi5 to have both attributes. Suppose we don't allow Swi5 to self-degrade since it has an inhibitor. How would this change affect the network's steady state behavior? We computed the attractor set for the cell-cycle TBN (Equation (1)) disallowing Swi5 to have the self-degradation property in Table 2. Compared to the attractor set with Swi5 self degrading (yellow loop) in Table 1A, the attractor set in Table 2 is bigger with 14 fixed points, half of which has Swi5 on. The attractor set in Table 1A is a subset of that in Table 2, meaning that the new attractors are due to Swi5 not degrading to 0. The biggest attractor in this new set is (0, 0, 0, 0, 1, 0, 1, 0, 0, 1, 0) which differs from the biggest attractor in Table 1A only by the presence of Swi5. This exercise has shown that slightly altering the degradation assumption dramatically affected the size and complexity of the cell-cycle's long term behavior.

**Table 2:** **The attractor set for the cell-cycle threshold Boolean network which does not contain Swi5's self-degradation property**.

**Basin size**	**Cln3**	**MBF**	**Clb5&6**	**Mcm1/SFF**	**Swi5**	**Cdc20&14**	**Cdh1**	**Cln1&2**	**SBF**	**Sic1**	**Clb1&2**
1383	0	0	0	0	1	0	1	0	0	1	0
380	0	1	0	0	1	0	0	1	1	1	0
139	0	0	0	0	1	0	0	1	1	1	0
108	0	1	0	0	1	0	1	0	0	1	0
10	0	0	0	0	1	0	0	0	0	1	0
8	0	0	0	0	0	0	0	1	1	0	0
6	0	1	0	0	1	0	0	0	0	1	0
5	0	0	0	0	0	0	0	0	0	0	0
4	0	0	0	0	1	0	0	1	1	0	0
1	0	0	0	0	0	0	1	0	0	0	0
1	0	0	0	0	0	0	0	0	0	1	0
1	0	1	0	0	0	0	0	0	0	1	0
1	0	1	0	0	0	0	1	0	0	1	0
1	0	0	0	0	0	0	1	0	0	1	0

*The results are based on setting the interaction coefficients a_g_ = −a_r_ = 1 and protein lifetime t_d_ = 1*.

As noted above, the only cycle constructed with all positive regulations in Figure 1 is between Clb1&2 and Mcm1/SFF, and this cycle is not sustained (both proteins are at state 0) in the network's long term behavior. To leave the cycle on indefinitely, that is, to keep Clb1&2 and Mcm1/SFF at state 1 perpetually, the sum of the interaction coefficients *a*_*g*_ associated with the positive regulations (green edges) must exceed the sum of *a*_*r*_ associated with the negative regulations (red edges) acting on Clb1&2. Since −*a*_*r*_ = *a*_*g*_ = 1, the cycle between Clb1&2 and Mcm1/SFF may get turned on, but does not endure. If this cycle is deleted, the network satisfies the hypothesis of Theorem 1. Because the cycle between Clb1&2 and Mcm1/SFF does not stay on, the network therefore yields a null attractor when all proteins are forced to self-degrade. Thus, following Theorem 2, the variety of fixed points in Table 1A is attributable to the 6 proteins with persistence (green self loop) and the cardinality of the attractor set satisfies the upper bound of 2^6^. Note that the fixed points in Table 1A differ at the proteins with persistence (green self loop), as predicted by Theorem 2. In Section 3.3, we present a network in which the cycle remains active in the steady state.

#### 3.2.3. Markovian self-degradation

Since self-degradation is not built into the Markovian transition functions of the TBN model in Equation (1), specifying incremental degradation is a cumbersome separate process that requires tracking each gene with the self-degradation property and counting the *t*_*d*_ time steps prior to a state change. More importantly, by not explicitly modeling degradation, the model in Equation (1) does not have the typical Boolean network behavior. In particular, a state can be repeated without the network having reached an attractor. For example, suppose we have a two member network in which the only regulations are: protein 1 positively regulates (green edge) protein 2, protein 1 self degrades (yellow loop), and protein 2 persists (green self loop). The interaction coefficient is *a*_21_ = 1. Further, suppose that a protein's lifetime is *t*_*d*_ = 2. Using the TBN of Equation (1), a network path is (1, 1) → (1, 1) → (0, 1). Markovianizing degradation via the following model eliminates this problem by augmenting the state space to express the degradation counter.

(4) $$x_{i}(t+1)=\{\begin{array}{ll} \;1, & \sum_{j}a_{ij}\text{I}(x_{j}(t)>0)>\alpha_{i}\\ \;0, & \sum_{j}a_{ij}\text{I}(x_{j}(t)>0)<\alpha_{i}\\ \text{max}(x_{i}(t)-\epsilon_{i},0), & \sum_{j}a_{ij}\text{I}(x_{j}(t)>0)=\alpha_{i} \end{array}$$

Here *I*(*x*_*j*_(*t*) > 0) is an expression indicator for protein *j*; ϵ_*i*_ ∈ [0, 1] is the degradation rate for protein *i*; all other parameters are as previously defined in Equations (1) and (2). Whether a protein degrades is determined by the degradation parameter ϵ_*i*_. A protein degrades quickly with a large value of ϵ_*i*_ and persists at ϵ_*i*_ = 0. The TBN model in Equation (1) with the protein lifetime parameter *t*_*d*_ = 1 is equivalent to setting ϵ = 1 for proteins with self-degradation (yellow loop) and ϵ = 0 for proteins with persistence (self green loop). Note that ϵ = 1/*t*_*d*_. Compared to the TBN model in Equation (1) for which self-degradation must modeled in a side process, Equation (4) explicitly models self-degradation as part of the TBN.

The third line in Equation (4) is meant solely as a device for Markovianizing degradation and persistence. Thus, *x*_*i*_(*t* + 1) ∈ [0, 1], but the regulatory relationships remain Boolean via the indicator *I*(*x*_*j*_(*t*) > 0). The state space has simply been augmented to allow self-degradation. A further modification that would bring a TBN model closer to a system of differential equations would be to eliminate *I*(*x*_*j*_(*t*) > 0) and allow node *j* to take state *x*_*j*_ ∈ [0, 1] in Equation (4).

So far self-degradation has been treated as a triggered event, i.e., decays occurs after the net influence on the protein is equal to the threshold. The model can be extended to have decay in the presence of a net regulatory effect (Hanel et al., 2012) by letting a protein be its own parent. The sums in Equation (4) would then include node *i* and line 3 could be omitted with < α_*i*_ replaced by ≤ α_*i*_. These extensions of Equation (4) need to be further studied to understand their properties and appropriateness for modeling a genetic regulatory network.

### 3.3. Sensitivity to interaction coefficient

To test the robustness of the cell-cycle TBN to different values of the interaction coefficient *a*_*ij*_, we changed the coefficient of the positive regulations (green edges) to *a*_*g*_ ∈ {2, 3}. The attractor sets associated with *a*_*r*_ = −1 and *a*_*g*_ = 2 and with *a*_*r*_ = −1 and *a*_*g*_ = 3 are in Tables 1B,C. The attractor set for the model with *a*_*r*_ = −1 and *a*_*g*_ = 2 is a subset, with different basin sizes, of the attractor set for the model with *a*_*r*_ = −1 and *a*_*g*_ = 1 (Table 1A). When *a*_*r*_ = −1 and *a*_*g*_ = 3, the network cycle between Clb1,2 and Mcm1/SFF is turned on indefinitely in the biggest attractor (0, 0, 0, 1, 1, 1, 1, 0, 0, 1, 1) which has a basin size of 1936 states. This is a consequence of positive regulations overcoming negative regulations acting on Clb1,2. With negative interactions fixed at *a*_*r*_ = −1, the attractor sets for networks with *a*_*g*_ > 3 are either identical or very similar to the set corresponding to *a*_*g*_ = 3 (Table 1C). For those attractor sets not identical with Table 1C, the main difference is the appearance of a two state attractor {(0, 0, 0, 1, 1, 1, 1, 0, 0, 1, 0), (0, 0, 0, 0, 1, 1, 1, 0, 0, 1, 1)}. This periodic attractor is very similar to the biggest fixed point in Table 1C because all the same proteins get turned on. The unequal attractor sets corresponding to different parameters indicate that the TBN model is not robust to variable interaction coefficients; the cell-cycle network exhibit different behaviors depending on the model specifications. Furthermore, certain parameter values sustain the network cycle between Clb1&2 and Mcm1/SFF and express cellular activities not previously seen.

Next we explored how increasing the degradation delay *t*_*d*_ changed the cell-cycle network's behavior. When we set −*a*_*r*_ = *a*_*g*_ = 1 and *t*_*d*_ > 1 in the cell-cycle TBN (Equation (1)) the same 7 attractors in Table 1A appear. Simulation results show that varying *a*_*r*_ and *a*_*g*_ with *t*_*d*_ yielded attractor sets that are sensitive only to the interaction coefficient.

### 3.4. Asynchronous gene response

The assumption that all genes in a network update simultaneously, *synchronous response*, may be too simplistic. For example, synchronous BoN models may yield attractors driven by the synchrony assumption (Ingerson and Buvel, 1984; Klemm and Bornholdt, 2005). While synchronous response is well-defined, asynchronous response has been defined and modeled in a variety of ways. One model of asynchrony works via an operator external to the BoN that randomly selects a subset of genes to update at each iteration while keeping the unselected genes constant (Ingerson and Buvel, 1984; Greil and Drossel, 2005; Skodawessely and Klemm, 2011). Another model of asynchrony is achieved by allowing different regulatory relationships to have different reaction rates (Thomas and D'Ari, 1990; Silvescu and Honavar, 2001; Shmulevich and Zhang, 2002). Unlike stochastic asynchrony, asynchrony due to varying reaction rates can be incorporated into a deterministic BoN. One type of deterministic asynchronous response can be modeled by allowing genes and proteins to have different self-degradation rates and different interaction coefficients *a*_*ij*_. A protein with a larger lifetime *t*_*d*_ in Equation (1) will take a longer time to reach state 0. Allowing different proteins to have different lifetimes imply different response times. A positive regulator with a higher interaction strength, |*a*_*ij*_|, can dominate a negative regulator with a smaller interaction strength and turn on the affected gene. Suppose in a four member network, the relationships {2 → 1, 3 → 1, 4 → 1} have the following attributes: *a*_12_ = −1, *a*_13_ = 1, *a*_14_ = 3. Compared to gene 3, gene 4 can neutralize the effect of the inhibitor gene 2 and turn on gene 1. In the absence of gene 4, gene 3 would not be able to turn on gene 1 if the inhibitor gene 2 is also on. In this perspective, the magnitude of the interaction, |*a*_*ij*_|, can be thought of as a rate. Assigning different interaction coefficients to proteins in a network may be a way to model asynchronous gene update. As we've discussed in Section 3.3, different choices of the coefficient may produce different attractor sets. More work is required to identify which attractors are insensitive to variable *a*_*ij*_ and their importance to the cell-cycle.

### 3.5. Synergy and antagonism

Thus far the TBN in Equation (1) assumes the regulatory effects are additive. However, some genes act together such that their combined effect is more or less than the sum of the individual effects. *Synergistic* regulation occurs when the joint effect of multiple parents is more than the sum of the individual effects. In contrast, *antagonistic* regulation results in a joint effect that is less than the sum of the individual effects. Such relationships have been studied in cancer cells in which genes exhibit a synergistic response to the combined effort of oncogenic mutations (McMurray et al., 2008). Since synergistic and antagonistic regulations can be critical to the function of a GRN, the interactions should be properly modeled. The TBN model in Equation (1)) can be extended to model these types of regulation by including the statistical interaction terms, ∑_*j,k*_ *a*_*i*(*jk*)_*x*_*j*_(*t*)*x*_*k*_(*t*), where the interaction coefficient *a*_*i*(*jk*)_ between parents *j* and *k* and child *i* are defined analogously to *a*_*ij*_. Synergy is represented by a positive *a*_*i*(*jk*)_ and antagonism by a negative *a*_*i*(*jk*)_. Interactions of order greater than two are similarly constructed.

## 4. Discussion

A TBN specified by linear functions and a threshold instead of truth tables is more quantitative at describing genetic regulatory network (GRN) dynamics. We illustrate how this framework can accommodate aspects of GRNs such as variable Markovian self-degradation, asynchronous gene update, and synergistic relationships. Furthermore, we found that the complexity of a GRN can be summarized by the presence of self-degradation and cycles comprised of only positive regulations. Although the model is more analytical compared to networks specified by truth tables, it still retains the qualitative interpretation of a BoN.

Inspection of the TBN model in Equation (1) to model the budding yeast cell-cycle showed that the attractor set relied on the assumptions of self-degradation and choice of interaction coefficient *a*_*ij*_. Changing these two aspects of the model changed the steady state behavior of the cell-cycle. Our extension of the TBN model using a threshold parameter as in Equation (2) permits greater flexibility in describing regulatory relationships. Another modification we suggested was Markovianizing degradation to facilitate incremental or delayed degradation. We also proposed varying the protein lifetime *t*_*d*_ and interaction coefficient among proteins to simulate asynchronous gene update and adding statistical interaction terms to account for synergistic effects.

Our theorems claimed that the composition of a TBN's attractor set depends on the presence and abundance of self-degradation (yellow loops), persistence (green self loops), and network cycles. Theorem 1 states that the null attractor is the only attractor for a network acyclic in the positive regulations (green edges) and in which all nodes self degrade. This result holds under varying interaction strength and degradation rates. Although the theorem was proved for TBNs, it applies to other Boolean network models that are not of the form in Equation (1) because the proof relies only on topological ordering in the positive regulations and self-degradation on all genes. Theorem 2 states that under a weaker definition of acyclicity, the complexity of the attractor structure is entirely determined by the configuration of persistent genes.

Future work includes characterizing the attractor set, e.g., determine an upper bound on its cardinality, for (a) the class of TBNs containing network cycles of positive regulations (green edges), and (b) the class of TBNs containing both persistence and network cycles of positive regulations in the presence of self-degradation and asynchronicity.

## Author contributions

Van Tran performed the majority of the analyses and primarily wrote the manuscript; Matthew N. McCall and Anthony Almudevar performed some analyses, wrote portions of the manuscript, and helped conceive the project; Helene R. McMurray provided biological expertise and helped conceive the project. All authors edited and approved the manuscript.

### Conflict of interest statement

The authors declare that the research was conducted in the absence of any commercial or financial relationships that could be construed as a potential conflict of interest.
